# Response of Grasshopper and Grasshopper Diversity to Different Grassland Types Under Enclosure Conditions

**DOI:** 10.1002/ece3.72704

**Published:** 2025-12-17

**Authors:** Chuanen Li, Xingmin Song, Mengjia Wang, Roman Jashenko, Jun Lin, Zhujun Cao, Huixia Liu, Rong Ji

**Affiliations:** ^1^ College of Life Science Xinjiang Normal University, Key Laboratory of Special Species and Regulatory Biology in Xinjiang, International Center for the Collaborative Management of Cross‐Border Pests in Central Asia Urumqi China; ^2^ International Center for the Collaborative Management of Cross‐Border Pests in Central Asia Xinjiang Normal University Urumqi China; ^3^ Tacheng, Research Field (Migratory Biology) Observation and Research Station of Xinjiang Tacheng China; ^4^ Institute of Zoology Ministry of Education and Science of Kazakhstan Almaty Kazakhstan; ^5^ Locust and Rodent Pest Prediction and Control Station of Xinjiang Urumqi China

**Keywords:** enclosure mode, locusts and grasshoppers diversity, response mechanism, west Tianshan Mountains in China

## Abstract

Enclosure is one of the important methods used to restore grassland ecosystems. Locusts and grasshoppers are important components of grassland ecosystems and can accurately reflect changes in their habitats. However, currently, the response in abundance of grassland locusts and grasshoppers within short‐term enclosure mode remains unclear. In this study, a 3‐year short‐term enclosure experiment on a natural grassland in Ili Kazak Autonomous Prefecture, Xinjiang, China, aimed to explore the response patterns of grassland locusts under fences. According to the results of this study, there were significant differences in the diversity of locusts and grasshoppers in various grassland types after short‐term captivity. Canonical Correlation Analysis shows that temperature is the main factor affecting grassland locusts at different altitudes and latitudes; annual precipitation and relative humidity are the main factors affecting the distribution of dominant locusts and grasshoppers in different grassland types; the duration of sunshine and the highest daily temperature are the main factors affecting the dominance of locusts and grasshoppers at different altitudes and latitudes. As altitude increased from the temperate desert steppes to the mountain meadows, vegetation cover presented a “decreasing‐increasing” trend, and the number of locusts and grasshoppers per unit quadrat showed a “decreasing‐increasing‐decreasing” trend. As a result of increased vegetation cover and altitude and decreased latitude, the community structure of locusts and grasshoppers shifted from a decrease in terricoles species such as the *Sphingonotus* species to an increase in phytophilous species like *Omocestus petraeus*. In contrast to the results of a study performed in the same area 40 years ago, this survey showed that four species of locusts and grasshoppers, including *Gomphocerus sibiricus,* shifted toward higher altitudes, causing changes in the community structure of locusts and grasshoppers at higher altitudes; *Stauroderus scalaris scalaris* replaced *Gomphocerus sibiricus* and evolved into a high‐altitude dominant species; and *Pararcyptera microptera microptera* shifted toward low altitudes. Therefore, in order to more accurately assess the restoration status of grassland ecosystems under fences, it is necessary to consider the diversity changes and influencing factors of locusts and grasshoppers, which may vary depending on different grassland types.

## Graphical abstract

We found that compared to 40 years ago, there has been a phenomenon of migration and diffusion of dominant locusts and grasshoppers, with some shifting toward higher altitudes and replacing the original dominant species, whereas others are shifting toward lower altitudes. We identified that temperature is the main factor affecting locusts and grasshoppers on different altitudes and latitudes, whereas precipitation is the main factor affecting locusts and grasshoppers on different types of grasslands. We found that the Indval analysis method was not entirely suitable for evaluating locusts and grasshoppers as indicators of grassland restoration under enclosure.

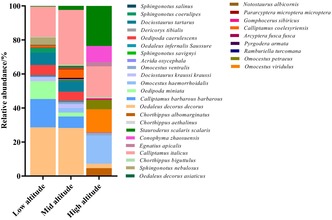

## 1 Introduction

Enclosure is a major driver of grassland health that affects many characteristics of these ecosystems, including vegetation, biomass, and biodiversity (McDonald et al. 2019). Enclosure promotes the natural restoration of species diversity in degraded grasslands by eliminating grazing pressure. The enclosure has increased the vegetation on the grassland, transforming it from a sedge/grassland community to a grassland/grassland community (Wei et al. 2017, 2021). The structural changes of arthropod communities in the enclosure grassland are related to the changes in vegetation types (Torma et al. 2023). The factors that affect insect diversity under enclosure or grazing may include enclosure period, grassland type, altitude, and latitude (Ma et al. 2017; Zhu et al. 2015). Many studies have shown that the effect of enclosure on insect diversity varies with specific circumstances (Torma et al. 2023). For example, the insect diversity of meadow steppes under enclosure was higher than that of dry grasslands, whereas in the alpine grasslands of Scotland, the insect diversity of sample plots under enclosure was higher than that of sample plots under grazing (Littlewood et al. 2012; Walcher et al. 2017). There was no significant difference in the insect diversity of desert steppes in Mongolia between sampling at three and 7 years, but a one year enclosure period was conducive to the maintenance of insect diversity (Wang et al. 2022). An experiment conducted in Hungary with alkaline meadow steppes and ordinary meadow steppes under short‐term enclosure showed that, compared with grazed steppes, enclosure caused the community structure of arthropods to shift toward hygrophilous species in the alkaline meadow steppes, but not in the ordinary meadow steppes (Torma et al. 2023). Other studies have revealed that changes in insect diversity under enclosure respond to altitude. For example, at altitudes of 700–1100 m, the response of Orthopterans in the German mountains to changes in environmental conditions varied across different steppe types. Compared with prairie meadows and moderately humid steppes, despite a strong increase in overall species richness in common pastures, neither the Community Farmland Index (CFI) nor the Community Temperature Index (CTI) had changed (Fumy et al. 2020). A study in Xizang shows that species richness and the Margalef richness index of grasshopper communities decreased significantly with increasing altitude, peaking at 1100–1600 m (Li et al. 2024). At altitudes of 800–1400 m, the diversity of arthropods in the Swiss mountains was positively correlated with vegetation structure (Wettstein and Schmid 1999), where at altitudes of 1350–3170 m, the increase in vegetation height in the sub‐alpine grasslands of the Alps positively affected the abundance and diversity of Orthopterans (Spalinger et al. 2012). From 1600 m to 2800 m in the Guandi Mountain, the insect community showed certain differentiation characteristics with the altitude gradient (Zhao et al. 2023). Compared with grazed grasslands, enclosures also increased the abundance of arthropods in the Scottish Highlands by 3–4 times (Littlewood et al. 2012).

Orthoptera shows a significant response to succession under grassland management (Fartmann et al. 2012). More and more studies are using insects as indicator species to assess the success of grassland restoration (Alignan et al. 2018). Orthopterans are sensitive indicators of grassland structure and health (Cherrill 2015). Locusts and grasshoppers, as the most common herbivorous insect species in grassland ecosystems, are closely related to their host plants and to insects of higher trophic levels. They play a crucial role in preserving the functions of grassland ecosystems by promoting material cycling and energy flow (Stout and Raubenheimer 2012). In this sense, locusts and grasshoppers can serve as an ideal insect taxon for revealing the underlying mechanisms of enclosure response and assessing the restoration of grassland structures (Schwarz and Fartmann 2022). Some studies have shown that grasshopper species richness did not differ between common pastures and ungrazed grasslands surrounding to the common pastures (Schwarz and Fartmann 2022). By contrast, the density of all of threatened species varied between common pastures and controls in all plots and within the two vegetation types with the highest grasshopper abundance, grasslands on mineral soil and fens (Schwarz and Fartmann 2022). Under long‐term enclosure, the community composition of locusts and grasshoppers underwent significant changes, with herbivorous locusts and grasshoppers (instead of omnivorous ones) becoming the dominant species (Jonas and Joern 2007). Unlike long‐term enclosures, the diversity of underground species in short‐term enclosures is higher than that in long‐term enclosures (Fenetahun et al. 2021). Other enclosure experiment results showed that female grasshopper's survival rate, mean survival time, and egg production were positively associated with increasing grazing intensities. The positive relationships are likely due to changes in plant nitrogen content and microhabitat induced by large herbivore grazing activities for locusts and grasshoppers (Zhu et al. 2020). An enclosure study on grasshopper assemblage response to seasonal grazing, rotational grazing, continuous resting, and continuous grazing was undertaken in the eastern Karoo, South Africa (Gebeyehu and Samways 2003). Rotationally grazed sites supported the highest number and abundance of grasshopper species, whereas continuously grazed sites had the lowest. Spring‐grazed and winter‐grazed sites were the most similar, with continuously rested sites being the next similar to these (Gebeyehu and Samways 2003). However, there is a lack of only consider short‐term enclosure, and comprehensively consider the effects of grassland type, altitude, and latitude separately on insect diversity within short‐term enclosure mode, and the differences in the response of locust and grasshopper diversity to short‐term enclosure (Báldi et al. 2013), as well as the related influencing factors, still need to be clarified (Wang et al. 2022).

To fill this knowledge gap, we have raised the following questions: (1) What are the differences in the diversity of locusts and grasshoppers in different grassland types, altitudes, and latitudes under the short‐term enclosure mode? (2) What are the key factors influencing the distribution of dominant grasshopper species after short‐term enclosure? (3) Is the Indicator Value (IndVal) analysis applicable when locusts and grasshoppers are used as indicator species for grassland restoration under short‐term enclosure? To validate these hypotheses, this study selected the Ili Kazakh Autonomous Prefecture of Xinjiang, China, as the study area and chose grasslands under a three‐year short‐term enclosure as sample plots.

## 2 Materials and Methods

### 2.1 Overview of the Study Area

**FIGURE 1:**
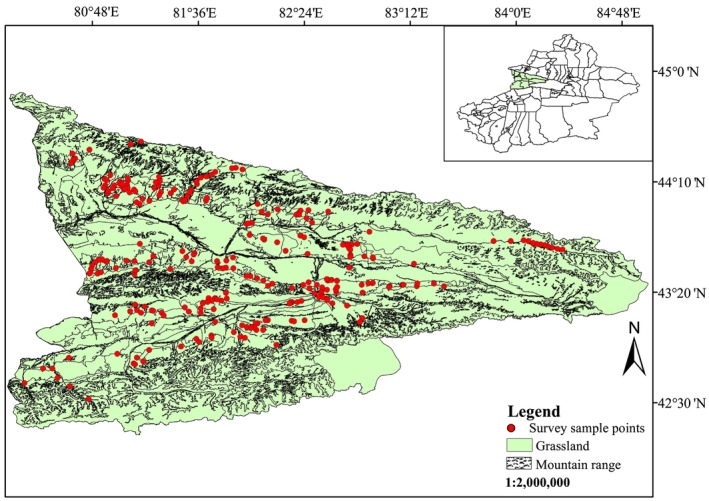
Sample points for the investigation of enclosed areas in the study area. This map is based on the standard map with the review number GS (2019) 1822 downloaded from the standard map service website of the National Bureau of Surveying, Mapping, and Geoinformation of China. The base map has not been modified.

The study area (Figure 1) is located in the northwest of Xinjiang, China, in a natural grassland area of 20.296 million hectares, accounting for 35.4% of the total grassland area in Xinjiang (Zhang, Fan, et al. 2022; Zhang, Wen, et al. 2022). Ili has a humid and mild climate, with rainy and moist mountains and rainless, dry plains. The annual average temperature is 10.5°C, and the annual average precipitation is 400–600 mm. There are a variety of grassland types, including lowland meadows, mountain meadows, temperate meadows, temperate steppes, temperate deserts, and temperate desert steppes (Zhang, Fan, et al. 2022; Zhang, Wen, et al. 2022). The grasslands in the study area have a significant vertical altitude distribution (500–4900 m), spanning across two latitudes (42°14′‐44°50′ E). The abundance of locusts and grasshoppers in this area, including dominant species, poses a significant threat to the local grassland ecology, agriculture, and animal husbandry, causing substantial losses (Li 2000).

### 2.2 Research Methods

#### 2.2.1 Design of the Enclosure Experiment

A 3‐year short‐term enclosure experiment (2021–2023) was conducted in the abovementioned study area after grassland mowing in 2020. During this period, only researchers were allowed to enter the experimental enclosure zone for survey and data collection. The zone also met the following conditions: (1) The zone was fenced using iron gauze to prevent the grazing of grass‐feeding livestock such as sheep, cows, and horses, as well as chickens and ducks, but free entry and exit of insects and other arthropods were allowed. (2) No one was allowed to enter the experimental enclosure zone to collect Chinese medicinal herbs or engage in other activities. To reduce the impact of mutual effects between grassland types, different altitudes, and latitudes on the experiment, representative grassland types were selected for each altitude segment in this study to set up sample plots. All plots in enclosed areas with an altitude of less than 1000 m are set up in temperate deserts, plots with an altitude of 1000–1800 m are set up in temperate steppes, and plots with an altitude of more than 1800 m are set up in temperate meadow steppes. The sample plots at various latitudes are set up in representative temperate deserts and intermediate altitudes sections.

The enclosure experiment included five different grassland types found in the study area (mountain meadows, temperate meadow steppes, temperate steppes, temperate deserts, and temperate desert steppes). The main vegetation in mountain meadows is *Dactylis glomerata* ; the main vegetation of temperate meadow steppes is *Ajania pallasiana* and *Poa annua* in Xinjiang; the main vegetation in temperate steppe is *Festuca ovina* and *Allium ramosum* ; the main vegetation in temperate deserts is *Seriphidium transilien*; the main vegetation in temperate desert steppes is *Stipa caucasica* and *Anabasis cretacea* (Xu et al. 1999). According to the division by the local forestry and grassland administration, the total enclosed area of the five grassland types amounted to 25.67 × 10^4^ m^2^. Three 50 × 150 m sample plots were set up in each grassland type, totaling 15 plots. Each sample plot was then demarcated with three 50 × 50 m quadrats, totaling 45 quadrats.

The method proposed by Tiede et al. (2018) was employed to divide the study area into three altitudinal gradients, namely, low altitudes (≤ 1000 m), intermediate altitudes (1000 m–1800 m), and high altitudes (≥ 1800 m). Three 50 × 150 m sample plots were set up for each altitude range, totaling nine. Each sample plot was then demarcated with three 50 × 50 m quadrats, totaling 27 quadrats.

Referring to Yuan's methods (Yuan and Chen 2009), the study area was divided into three latitudinal gradients at intervals of 0.67° and 74.37 km, namely, low latitudes (≤ 43.21 N), intermediate latitudes (43.21–43.86 N), and high latitudes (≥ 43.86 N). Again, three 50 × 150 m sample plots were set up for each latitude range, totaling nine. Each sample plot was demarcated with three 50 × 50 m quadrats, so there were 27 quadrats in total.

#### 2.2.2 Survey Methods and Data Sources

During the insect growth season (June to August) in 2021–2023, data on locusts and grasshoppers, vegetation, terrain, landform, and other information were collected and recorded at the test site. The field surveys in 2022 and 2023 were conducted on random quadrats, where the sample plots were randomly selected from the sample plots set up in advance, and were kept at least 5 m away from the fence to reduce data errors caused by the free entry and exit of insects through the fence. Targeted surveys were conducted in the first year, followed by supplemental surveys in the second and third years on the basis of the sampling points from the first year.

Line transects 50 m in length were set up at intervals of 10 m in each sample plot following the line transect method, totaling five. The species and quantities of locusts and grasshoppers were surveyed using the sweeping method. Sweeping was performed along each line transect at a speed of 1.8 km/h, and the species and quantities of locusts and grasshoppers within a 1.5 m scope of each line transect on both sides were recorded (He 2021). Longitude, latitude, altitude, slope, aspect, and other information were recorded as well. Each line transect was swept 100 times. Species difficult to identify in the field were marked and brought back for specimen preparation and identification. The height, fresh weight, density, and other information of vegetation within each sample plot were surveyed using the five‐point sampling method (Guo et al. 2023). During the sampling in 2022 and 2023, a total of 387 quadrats were surveyed for different grassland types, altitudes, and latitudes. Data were obtained on locusts and grasshoppers from 1935 sampling points and on vegetation from 387 quadrats and 1935 sampling points.

Additionally, meteorological data, including temperature and precipitation from 10 Chinese standard meteorological stations within the study area, were obtained for the study years (Table A1). A vector map of China with a scale of 1:50,000 was downloaded from the National Fundamental Geographic Information System (NFGIS) for terrain and landform data.

#### 2.2.3 Data Processing and Analysis

Orthopteran species whose individual number accounted for > 10% of the total number of individuals in a sample plot were defined as a dominant species. Similarly, a species whose individual number accounted for ≥ 1% but ≤ 10% of the total number of individuals in a sample plot was defined as a common species, and species whose individual number accounted for < 1% of the total number of individuals in a sample plot were defined as a rare species (Wang et al. 1996; Xu et al. 1999). During the field survey, a few locust and grasshopper nymphs were collected, but their main morphological features had not yet fully developed, and they could not be identified to species level. Thus, unless otherwise specified, this study only involved adult locusts and grasshoppers.

A generalized linear model (GLM) was employed to explore the mixed effects of grassland type, altitude, and latitude on grasshopper diversity. Canonical correlation analysis (CCA) was performed to identify the main ecological factors affecting changes in dominant locusts and grasshoppers within short‐term enclosure mode. Autocorrelation analysis was conducted on ecological factors using the Spearman correlation analysis, where ecological factors with absolute correlation coefficients > 0.8 and low contribution degrees were removed to reduce errors caused by interactions between ecological factors (Benesty et al. 2009).

This article uses the indicator species analysis method (IndVal, indicator value) proposed by Dufrêne and Legendre (1997) to evaluate whether locusts are suitable as indicators of grassland restoration after enclosure. IndVal analysis is widely used in research on species indication, such as the survey on the biodiversity of arthropods after forest fires in the Southern Alps (Moretti et al. 2004) and research on the fire‐induced changes in the classification and function of humic beetle communities in fire‐sensitive areas of the central Alps (Moretti et al. 2010). This index is maximum when all individuals of a species are found in a single group of sites and when the species occurs in all sites of that group; it is a symmetric indicator. The statistical significance of the species indicator values is evaluated using a randomization procedure. Contrary to TWINSPAN, the indicator index for a given species is independent of the other species relative abundances, and there is no need to use pseudospecies, so that the heterogeneity of the species combination observed at any location can be well preserved. The R package, labdsv2.1–0 (Roberts 2016), was used for this analysis, and species with an indicator value > 0.25 were taken as the indicator species in that area.

We use ArcGIS (10.8) to draw sample maps, R (4.3.1), and SPSS (25.0) for diversity and indicative species analysis.

## 3 Results

### 3.1 Distribution of Locusts and Grasshoppers Species in the Research Area

A total of 16,896 locusts and grasshoppers were collected in the enclosed area, and they belonged to five families, 19 genera, and 32 species. Analyzing the data and discovering, among the five grassland types, temperate desert steppes boasted the largest number of locust and grasshopper species, whereas temperate steppes had the lowest. After short‐term enclosure, the intermediate‐altitude range boasted the largest number of locust and grasshopper species, whereas the high‐altitude range had the smallest. The intermediate‐altitude range also had the highest density of locusts and grasshoppers in quadrats. The intermediate‐latitude range boasted the largest number of locust and grasshopper species, whereas the low‐latitude range had the smallest. The high‐latitude range had the highest density of locusts and grasshoppers in quadrats.

**FIGURE 2:**
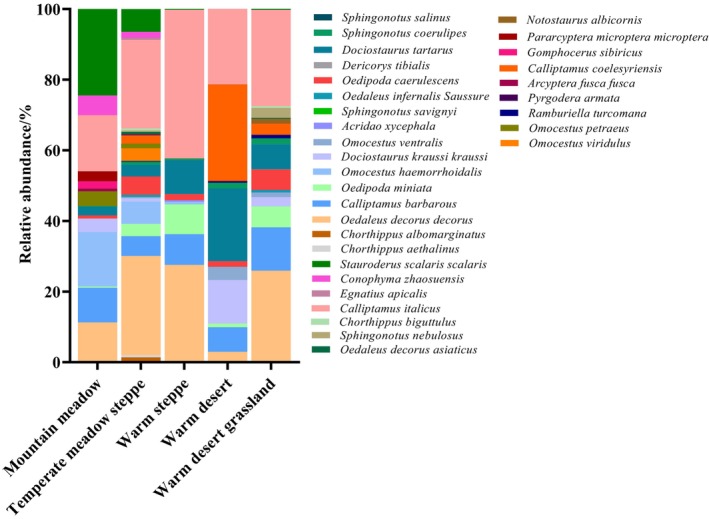
Relative abundance of grassland locusts and grasshoppers in short‐term enclosure areas of different grassland types.

The dominant locust and grasshopper species in the mountain meadows (1600–2600 m) were *Oedaleus decorus decorus*, *Omocestus haemorrhoidalis*, *Calliptamus italicus*, and *Stauroderus scalaris scalaris*. The dominant locust and grasshopper species in the temperate meadow steppes (1800–2100 m) and the temperate steppes (1100–1600 m) were *O. decorus decorus* and *C. italicus* . The dominant locust and grasshopper species in the temperate deserts (600–1400 m) were *Dociostaurus kraussi kraussi*, *C. coelesyriensis*, and *C. italicus* . The dominant locust and grasshopper species in the temperate desert steppes (1100–1700 m) were *O. decorus decorus*, *C. barbarous barbarous*, and *C. italicus* . *C. italicus* was found in each grassland type and was a dominant species in each. *O. decorus decorus* was also a dominant species in all grassland types except for the temperate deserts. *Arcyptera fusca fusca* was found only in the mountain meadows as a common species, whereas *Notostaurus albicornis* was present only in the temperate desert steppes as a rare species (Figure 2).

**FIGURE 3:**
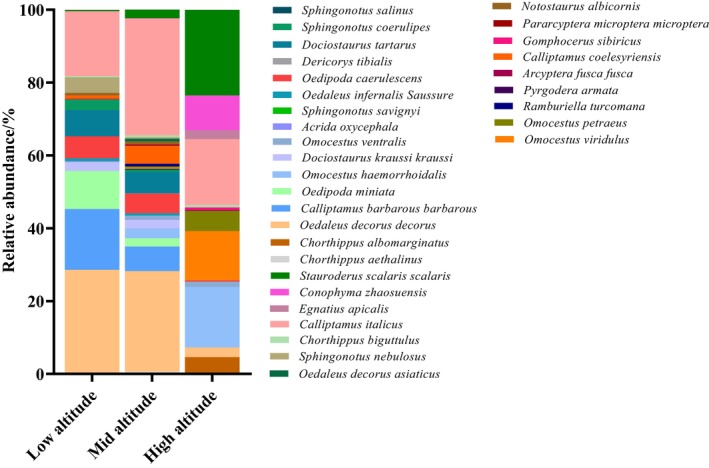
Relative abundance of grassland locusts and grasshoppers in short‐term enclosure areas at different altitudes.

The low‐altitude range (< 1000 m) had four dominant locust and grasshopper species: *O. decorus decorus*, *C. barbarous barbarous*, *Oedipoda miniata*, and *C. italicus* ; five common species: *D. kraussi kraussi*, *O. caerulescens* , *D. tartarus*, *Sphingonotus coerulipes*, and *S. nebulosus* ; and 13 rare species: *O. ventralis* , *Acrida oxycephala*, *Sphingonotus savignyi*, *O. infernalis Saussure*, *S. salinus* , *Omocestus viridulus*, *Ramburiella turcomana*, *Pyrgodera armata*, *C. coelesyriensis*, *N. albicornis*, *Chorthippus biguttulus*, *Egnatius apicalis*, and *S. scalaris scalaris* . The intermediate‐altitude range (1000–1800 m) had two dominant locust and grasshopper species: *O. decorus decorus* and *C. italicus* ; nine common species: *C. barbarous barbarous*, *O. miniata* , *O. haemorrhoidalis* , *D. kraussi kraussi*, *O. ventralis* , *O. caerulescens* , *D. tartarus*, *C. coelesyriensis*, and *S. scalaris scalaris* ; and 20 rare species: *Chorthippus albomarginatus*, *C. aethalinus*, *O. infernalis Saussure*, *Dericorys tibialis*, *S. coerulipes*, *S. salinus* , *O. viridulus*, *Omocestus petraeus*, *R. turcomana*, *P. armata* , *A. fusca fusca* , *Gomphocerus sibiricus*, *Pararcyptera microptera microptera*, *N. albicornis*, *Chrysochraon dispar major*, *S. nebulosus* , *C. biguttulus* , *E. apicalis*, and *Conophyma zhaosuensis*. The high‐altitude range (> 1800 m) had four dominant locust and grasshopper species: *O. haemorrhoidalis* , *O. viridulus*, *C. italicus* , and *S. scalaris scalaris* ; six common species: *C. albomarginatus* , *O. decorus decorus*, *O. ventralis* , *O. petraeus*, *E. apicalis* , and *C. zhaosuensis*; and four rare species: *O. caerulescens* , *A. fusca fusca* , *G. sibiricus* , and *C. biguttulus* . *C. italicus* was present at each altitude as a dominant species. *O. decorus decorus* was a dominant species at low and intermediate altitudes, whereas *A. oxycephala* and *S. savignyi* appeared only at low altitudes as common species. *C. aethalinus*, *A. fusca fusca* , *P. microptera microptera*, and *C. dispar major* were only present at intermediate altitudes and as rare species (Figure 3).

The low‐latitude range (< 43.21 N) had three dominant locust and grasshopper species, namely *O. decorus decorus*, *C. italicus* , and *S. scalaris scalaris* ; six common species, including *C. albomarginatus* , *C. barbarous barbarous*, *O. caerulescens* , *O. petraeus*, and *P. microptera microptera*; and three rare species, namely *O. miniata* , *O. viridulus*, and *A. fusca fusca* . The intermediate‐latitude range (43.21–43.86 N) had two dominant locust and grasshopper species, namely *O. decorus decorus* and *C. italicus* ; 11 common species, namely *C. barbarous barbarous*, *O. miniata* , *O. haemorrhoidalis* , *D. kraussi kraussi*, *O. caerulescens* , *D. tartarus*, *O. viridulus*, *C. coelesyriensis*, *S. nebulosus* , *C. zhaosuensis*, and *S. scalaris scalaris* ; and 16 rare species, including *C. albomarginatus* , *C. aethalinus*, *O. ventralis* , *S. savignyi* , *O. infernalis Saussure*, *D. tibialis* , *S. coerulipes*, *S. salinus* , *O. petraeus*, *C. coelesyriensis*, *G. sibiricus* , *P. microptera microptera*, *S. nebulosus* , *C. biguttulus* , and *E. apicalis* .

**FIGURE 4:**
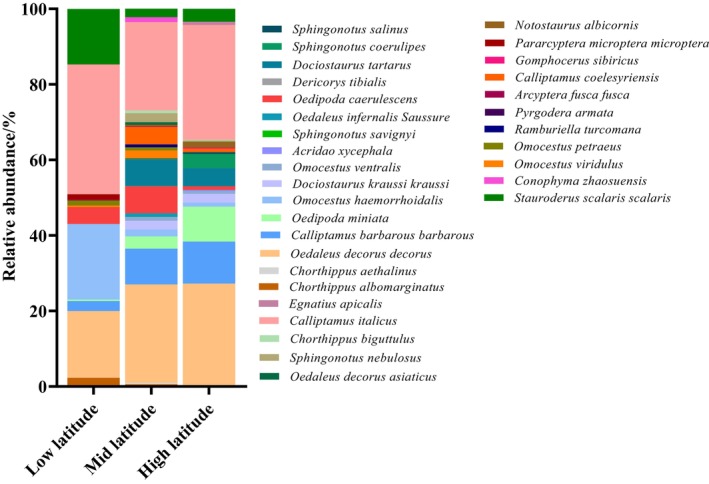
Relative abundance of grassland locusts and grasshoppers in short‐term enclosure areas at different latitudes.

The high‐latitude range (> 43.86 N) had three dominant locust and grasshopper species, namely *O. decorus decorus*, *C. barbarous barbarous*, and *C. italicus* ; seven common species, namely *O. miniata* , *O. haemorrhoidalis* , *D. kraussi kraussi*, *D. tartarus*, *S. coerulipes*, *N. albicornis*, and *S. scalaris scalaris* ; as well as 12 rare species, including *O. ventralis* , *A. oxycephala* , *O. caerulescens* , *S. salinus* , *O. petraeus*, *C. coelesyriensis*, *G. sibiricus* , *P. microptera microptera*, *S. nebulosus* , *C. biguttulus* , and *E. apicalis* . *O. decorus decorus* and *C. italicus* were dominant species at each of the three latitudinal gradients. *C. aethalinus*, *S. savignyi* , *D. tibialis* , *R. turcomana*, *P. armata* , *C. dispar major*, and *C. zhaosuensis* were found only at intermediate latitudes, whereas *A. oxycephala* was discovered only at high latitudes (Figure 4).

### 3.2 Changes in Dominant Locusts and Grasshoppers in Mixed Effects

GLM analysis revealed that, although controlling for other variables, the effects of grassland type, altitude, and latitude on grasshopper diversity indices differed significantly (Table 1). Details are as follows: Shannon index: Compared to mountain meadows, the Shannon index increased across all grassland types by 55.20%, 5.30%, 33.80%, and 11.80%, respectively. A significant difference (*p* < 0.05) was observed between temperate desert steppe and mountain meadows. Compared to high altitude, the Shannon index decreased by 6.70% at low altitude (non‐significant) but increased significantly by 13.5% at mid‐altitude (*p* < 0.05). Compared to high latitudes, the Shannon index decreased by 8.10% and 24.30% at low and mid‐latitudes, respectively. A significant difference (*p* < 0.05) was found only between mid‐ and high‐latitude regions.

**TABLE 1:** Mixed effects of locust diversity under three comprehensive conditions of grassland type, altitude, and latitude.

Diversity index	Reference level	Compare level	Estimate	*p*
Shannon–Wiener index	High altitude	Low altitude	0.943	0.363
		Mid altitude	1.135	0.001**
	High latitude	Low latitude	0.918	0.153
		Mid latitude	0.757	0.000**
	Mountain meadow	Temperate meadow grassland	1.552	0.000**
		Warm desert	1.053	0.672
		Warm desert grassland	1.388	0.000**
		Warm grassland	1.118	0.339
Simpson index	High altitude	Low altitude	1.003	0.808
		Mid altitude	0.973	0.000**
	High latitude	Low latitude	0.990	0.408
		Mid latitude	1.013	0.210
	Mountain meadow	Temperate meadow grassland	0.995	0.749
		Warm desert	0.976	0.313
		Warm desert grassland	0.990	0.533
		Warm grassland	0.972	0.226
Margalef richness index	High altitude	Low altitude	1.065	0.000**
		Mid altitude	1.024	0.248
	High latitude	Low latitude	0.835	0.463
		Mid latitude	1.058	0.000**
	Mountain meadow	Temperate meadow grassland	0.912	0.186
		Warm desert	1.541	0.145
		Warm desert grassland	1.195	0.008**
		Warm grassland	0.949	0.606
Berger–Parker index	High altitude	Low altitude	1.074	0.569
		Mid altitude	0.882	0.096
	High latitude	Low latitude	0.663	0.000**
		Mid latitude	0.658	0.000**
	Mountain meadow	Temperate meadow grassland	0.792	0.098
		Warm desert	0.857	0.519
		Warm desert grassland	0.740	0.045*
		Warm grassland	0.556	0.012*
Pielou uniformity index	High altitude	Low altitude	0.934	0.425
		Mid altitude	1.123	0.023*
	High latitude	Low latitude	0.954	0.555
		Mid latitude	0.724	0.000**
	Mountain meadow	Temperate meadow grassland	1.730	0.000**
		Warm desert	0.848	0.315
		Warm desert grassland	1.381	0.002**
		Warm grassland	1.250	0.155

* Indicates a significant impact (*p* < 0.05).

** Indicates an extremely significant impact (*p* < 0.01).

*Simpson index*: Compared to mountain meadows, the Simpson index decreased by 0.50%, 2.40%, 1.00%, and 2.80% across grassland types, with no significant differences. The Simpson index increased by 0.30% at low altitude (non‐significant) but decreased significantly by 2.70% at mid‐altitude (*p* < 0.05). The Simpson index decreased by 0.10% and 0.05% at low and mid‐latitudes, respectively, with no significant differences.

*Margalef richness index*: Compared to mountain meadows, the richness index decreased by 8.80% and 5.10% in temperate meadow steppe and temperate steppe, respectively, but increased by 54.10% and 19.50% in temperate desert and temperate desert steppe. A significant difference (*p* < 0.05) was observed only for temperate desert steppe. Altitude effect: Compared to high altitude, the richness index increased by 6.50% and 2.40% at low and mid‐altitudes, respectively, with a significant difference at low altitude (*p* < 0.05). Compared to high latitudes, the richness index decreased by 16.50% at low latitudes but increased significantly by 5.80% at mid‐latitudes (*p* < 0.05).

*Parker index*: The Parker index decreased by 20.80%, 14.30%, 26.00%, and 44.40% in temperate meadow steppe, temperate desert, temperate desert steppe, and temperate steppe, respectively. Significant differences (*p* < 0.05) were observed between temperate desert/temperate desert steppe and mountain meadows. The Parker index increased by 7.40% at low altitude and decreased by 11.80% at mid‐altitude, with no significant differences compared to high altitude (*p* < 0.05). The Parker index decreased significantly by 33.70% and 34.20% at low and mid‐latitudes, respectively (*p* < 0.05).

*Pielou index*: The Pielou index decreased by 15.20% in the temperate desert but increased by 73.00%, 38.10%, and 25.00% in the temperate meadow steppe, temperate desert, and temperate steppe, respectively. Significant differences (*p* < 0.05) were observed between temperate meadow steppe/temperate desert steppe and mountain meadows. The index decreased by 6.40% at low altitude but increased significantly by 12.30% at mid‐altitude (*p* < 0.05). The index decreased by 4.60% and 27.60% at low and mid‐latitudes, respectively, with a significant difference between mid‐ and high‐latitude regions (*p* < 0.05).

### 3.3 Main Ecological Factors Affecting Changes in Dominant Locust and Grasshopper Species

**FIGURE 5:**
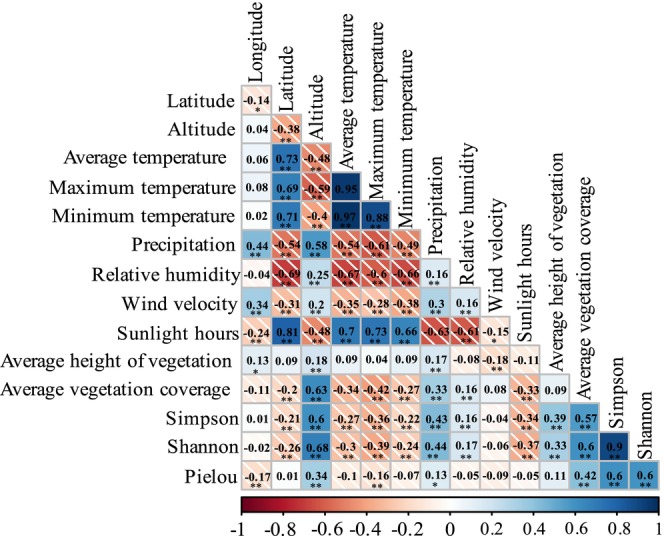
Autocorrelation analysis between environmental factors. The red diagonal square indicates a negative correlation between factors; the blue square indicates a positive correlation between factors. The depth of color indicates the strength of correlation; the value represents the correlation coefficient. * indicates significant impact (*p* < 0.05); ** indicates extremely significant impact (*p* < 0.01).

**FIGURE 6:**
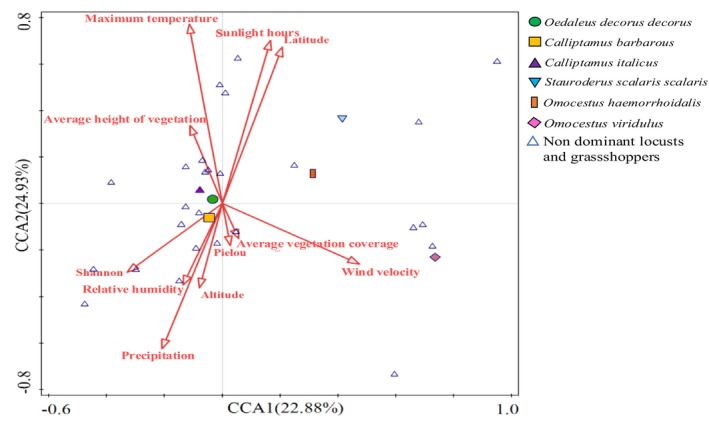
CCA (Canonical correlation analysis) of dominant locusts and grasshoppers and environmental factors under different grassland types. Symbols represent corresponding species; the same below.

Autocorrelation analysis was performed on 15 environmental factors that potentially affect change in the diversity of dominant locusts and grasshoppers, including longitude, latitude, altitude, daily average temperature, daily maximum temperature, daily minimum temperature, annual precipitation, relative humidity, wind speed, sunshine duration, average vegetation height, average vegetation cover, vegetation diversity, and vegetation evenness (Figure 5). Factors with absolute correlation coefficients > 0.8 were screened, and those with high contribution degrees were retained for the CCA. The results showed that, within short‐term enclosure mode, the contribution degrees of sunshine duration, wind speed, daily maximum temperature, and annual precipitation to the diversity of the dominant locusts and grasshoppers in the different grassland types were > 10% and highly significant (*p* < 0.01). The contribution degrees of relative humidity, average vegetation cover, and latitude were > 5% and were also significant (*p* < 0.05) (Figure 6 and Table 2).

**TABLE 2:** Contribution and significance of environmental factors under different grassland types on dominant locusts and grasshoppers.

Ecological factors	Explains %	Contribution %	pseudo‐F	*p*
Sunlight hours	1.1	15.0	3.0	0.002**
Wind velocity	1.2	16.0	3.3	0.002**
Maximum temperature	0.9	12.7	2.6	0.002**
Precipitation	0.8	10.8	2.2	0.004**
Relative humidity	0.7	9.3	1.9	0.016*
Average vegetation coverage	0.7	9.1	1.9	0.004**
Latitude	0.6	7.8	1.6	0.05*
Shannon index	0.4	5.5	1.1	0.254
Average height of vegetation	0.4	5.4	1.1	0.288
Pielou uniformity index	0.3	4.5	0.9	0.51
Altitude	0.3	4.0	0.8	0.638

* Indicates a significant impact (*p* < 0.05).

** Indicates an extremely significant impact (*p* < 0.01), same below.

**FIGURE 7:**
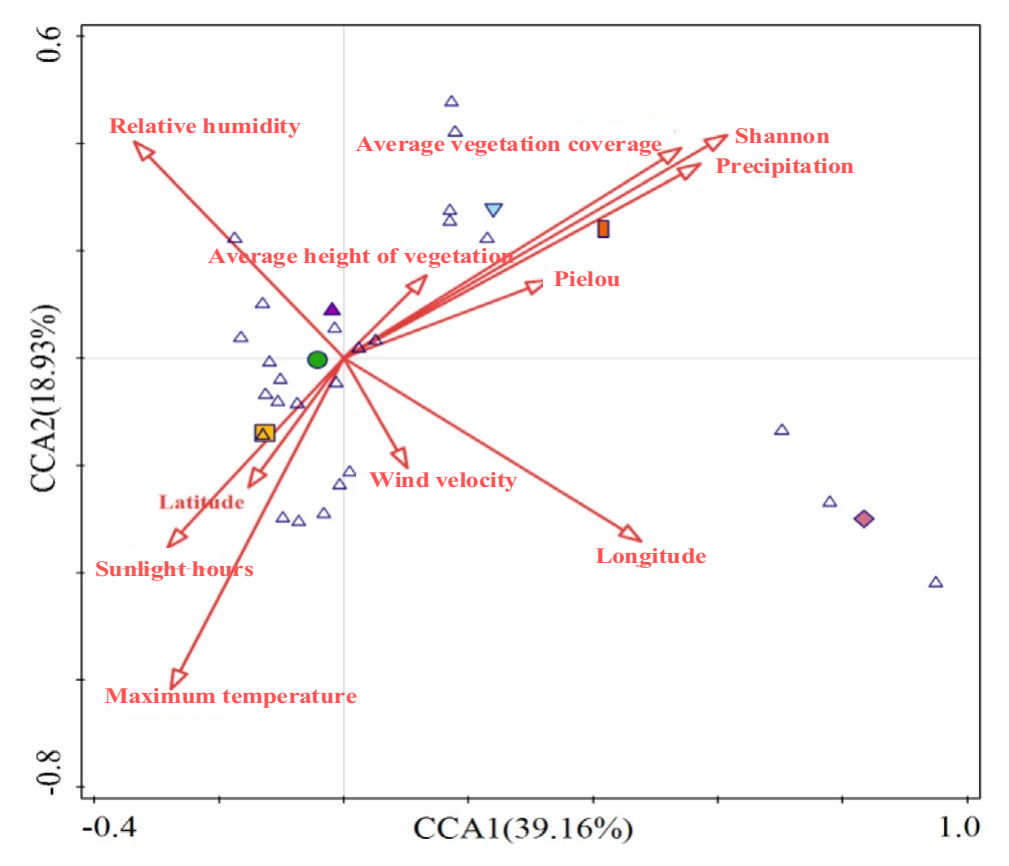
CCA of dominant locusts and grasshoppers and environmental factors under different altitudes.

Vegetation diversity, longitude, annual precipitation, relative humidity, daily maximum temperature, sunshine duration, latitude, and average vegetation cover exerted highly significant effects on the diversity of the dominant locusts and grasshoppers at different altitudes (*p* < 0.01), whereas wind speed and vegetation evenness significantly affected the diversity of dominant locusts and grasshoppers (*p* < 0.05). Specifically, the contribution degrees of vegetation diversity, longitude, annual precipitation, and daily maximum temperature to the diversity of the dominant locusts and grasshoppers were > 10%. Vegetation diversity, with a contribution degree of 19.3%, was the main factor affecting the diversity of the dominant locusts and grasshoppers within the short‐term enclosure mode (Figure 7 and Table 3).

**TABLE 3:** Contribution and significance of environmental factors under different altitudes on dominant locusts and grasshoppers.

Ecological factors	Explains %	Contribution %	pseudo‐F	*p*
Shannon index	3.0	19.3	8.2	0.002**
Longitude	2.5	16.4	7.2	0.002**
Precipitation	1.5	10.1	4.5	0.002**
Relative humidity	1.2	7.6	3.4	0.002**
Maximum temperature	2.3	15.1	6.9	0.002**
Wind velocity	0.8	5.5	2.5	0.018*
Sunlight hours	0.9	5.8	2.7	0.002**
Latitude	1.1	7.2	3.4	0.002**
Average height of vegetation	0.7	4.4	2.1	0.102
Average vegetation coverage	0.7	4.9	2.3	0.002**
Pielou uniformity index	0.6	3.7	1.7	0.028*

* Indicates a significant impact (*p* < 0.05).

** Indicates an extremely significant impact (*p* < 0.01), same below.

**FIGURE 8:**
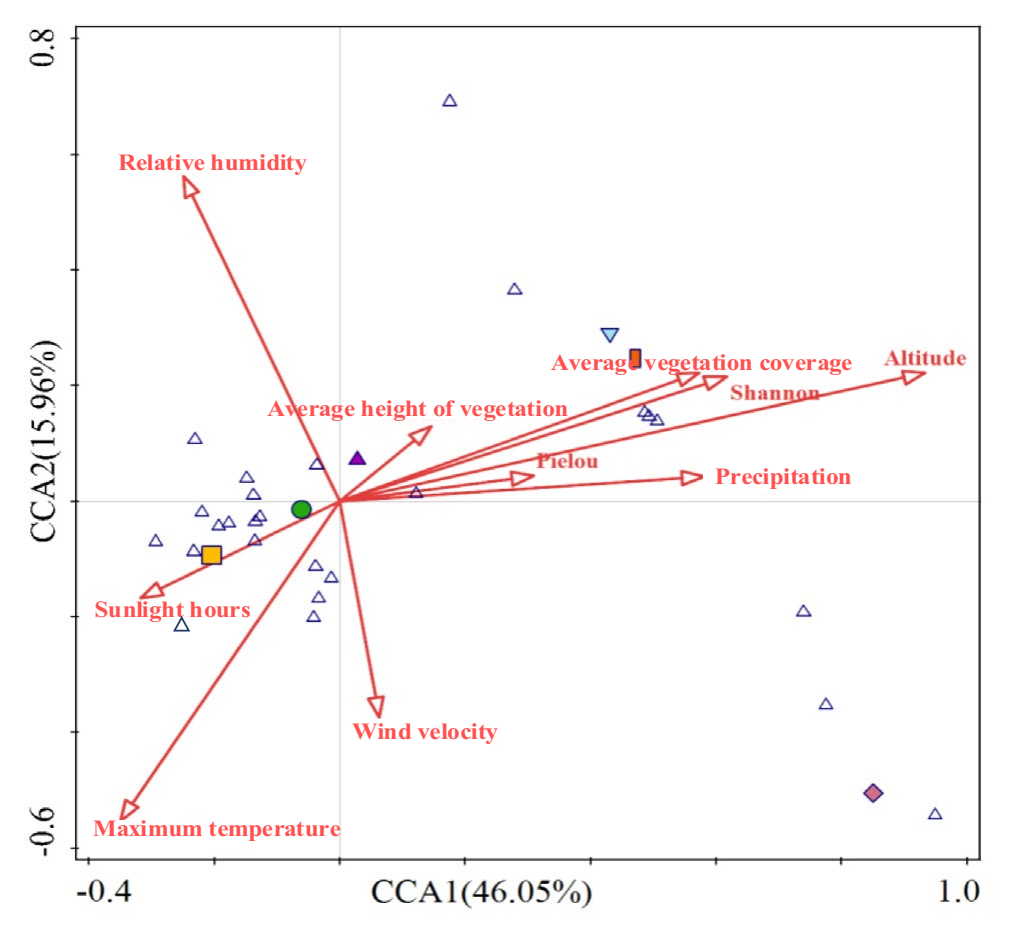
CCA of dominant locusts and grasshoppers and environmental factors under different latitudes.

Within short‐term enclosure mode, altitude, relative humidity, precipitation, sunshine duration, daily maximum temperature, and wind speed had highly significant effects on the diversity of dominant locusts and grasshoppers at different latitudes (*p* < 0.01), whereas vegetation evenness significantly affected the diversity of the dominant locusts and grasshoppers (*p* < 0.05). Specifically, the contribution degrees of altitude and relative humidity to the diversity of the dominant locusts and grasshoppers at different latitudes were > 10%. Altitude, with a contribution degree of 41.3%, was the main factor affecting the diversity of the dominant locusts and grasshoppers within the short‐term enclosure mode (Figure 8 and Table 4).

**TABLE 4:** Contribution and significance of environmental factors under different latitudes on dominant locusts and grasshoppers.

Ecological factors	Explains %	Contribution %	pseudo‐F	*p*
Altitude	5.9	41.3	16.9	0.002**
Relative humidity	1.6	11.1	4.6	0.002**
Precipitation	1.2	8.3	3.5	0.002**
Sunlight hours	1.3	9.3	3.9	0.002**
Maximum temperature	1.1	8.0	3.4	0.002**
Wind velocity	1.1	7.5	3.2	0.002**
Average height of vegetation	0.6	4.5	1.9	0.134
Pielou uniformity index	0.6	4.3	1.9	0.044*
Shannon index	0.5	3.2	1.4	0.1
Average vegetation coverage	0.3	2.4	1.0	0.448

* Indicates a significant impact (*p* < 0.05).

** Indicates an extremely significant impact (*p* < 0.01), same below.

### 3.4 The Applicability of Locusts and Grasshoppers as Indicator Species for Grassland Restoration

**FIGURE 9:**
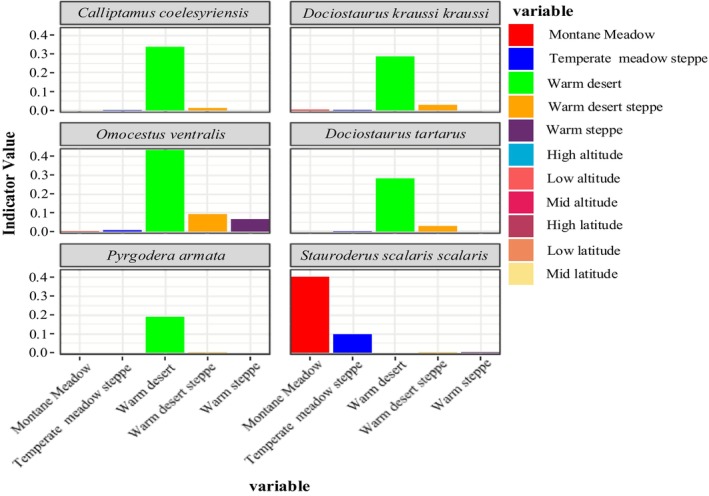
Indicator species and their indicator values under different grassland types.

**FIGURE 10:**
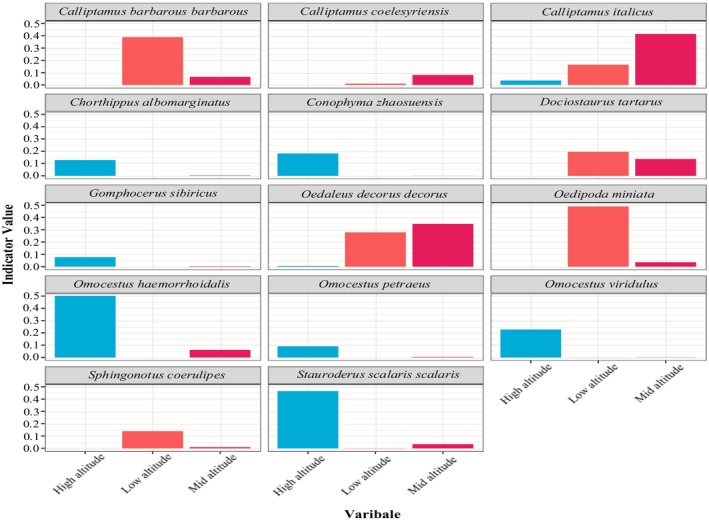
Indicator species and their indicator values under different altitudes.

**FIGURE 11:**
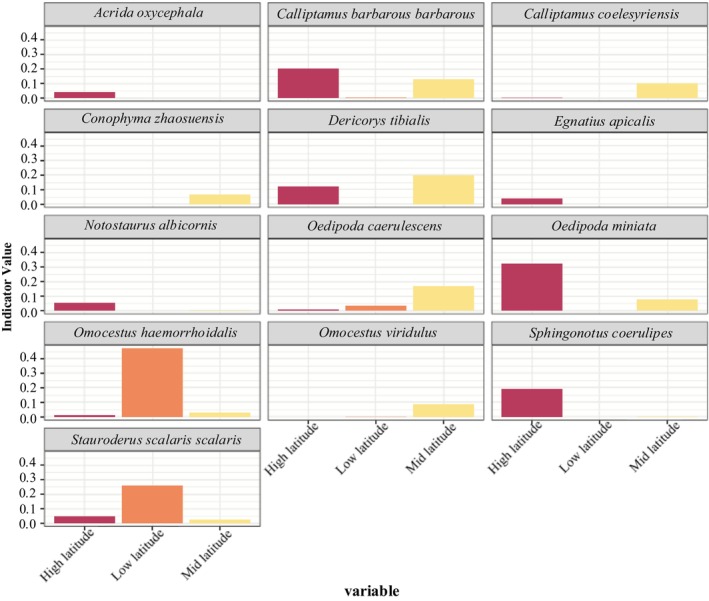
Indicator species and their indicator values under different latitudes.

Indicator species are biological indicators of habitat types or combinations of habitat types (Lawton and Gaston 2001). According to the results of the IndVal analysis under different conditions in the study area, *S. scalaris scalaris* was an indicator species for the mountain meadows, *D. tartarus* was an indicator species for the temperate deserts (Figure 9), and there were no indicator species for the temperate meadow steppes, temperate steppes, or temperate desert steppes (Table A2). *C. italicus* was an indicator species at low altitudes, *O. miniata* was an indicator species at intermediate altitudes, and *O. haemorrhoidalis* and *S. scalaris scalaris* were indicator species at high altitudes (Figure 10 and Table A3). *O. haemorrhoidalis* was an indicator species at middle latitudes (Figure 11), and there were no indicator species at low or high latitudes (Table A4). The theoretical calculation results were not entirely consistent with the field survey results.

## 4 Discussion

### 4.1 Diversity of Locusts and Grasshoppers in Response to Short‐Term Enclosure Mode and Main Influencing Factors

Our results showed that there were differences in the diversity of grassland locusts and grasshoppers under different experimental conditions during enclosure, which validates H1. Grasshopper diversity in both temperate meadow steppe and temperate desert steppe was significantly higher than in mountain meadows. The temperate meadow steppe exhibited intermediate average vegetation coverage and height between mountain meadows and temperate steppe, creating a stable microclimate and abundant resources that support a diverse grasshopper community (Bai et al. 2007). In the temperate desert steppe, although grazing exclusion affected forage yield (both dry and fresh), vegetation height, and coverage, the arid environment remained relatively unchanged, fostering a unique diversity of ground‐dwelling grasshopper species adapted to this habitat. Mid‐altitude regions harbored more grasshopper species but showed significantly lower diversity indices compared to high‐altitude areas. This may be attributed to intensified human activities or environmental stressors (e.g., altered temperature and precipitation patterns) at mid‐altitudes (Rahbek 2004; Sui et al. 2024). In contrast, mid‐latitude regions exhibited significantly higher diversity than high‐latitude regions, likely because of their location in low‐lying valley areas within the study basin, where more favorable temperature and precipitation conditions support greater biodiversity (Gaston 2000; Zhang et al. 2024). Thus, the lower diversity at mid‐altitudes may reflect a “human activity gradient effect,” where mid‐altitude zones are more susceptible to land use changes (Sala et al. 2000). Meanwhile, the high diversity observed in mid‐latitude regions and temperate meadow steppe aligns with the *Intermediate Disturbance Hypothesis (IDH)* (Connell 1978), suggesting that moderate environmental pressures promote species coexistence.

*C. italicus* , *O. decorus decorus*, *C. barbarous barbarous*, *O. miniata* , *D. tartarus*, and *S. coerulipes* were found in each grassland type, and among them, *C. italicus* and *O. decorus decorus* were both dominant species. However, each grassland type was particularly unique in its grasshopper and locust species composition. For example, *A. fusca fusca* was found only in the mountain meadows, and the presence of *C. albomarginatus* , *C. aethalinus*, *S. savignyi* , and *O. viridulus* was limited to the temperate meadow steppes, whereas *D. tibialis* and *N. albicornis* were found only in the temperate desert steppes. This may be related to the individual size and biotype of locusts and grasshoppers, vegetation cover, etc. Research has shown that species of smaller size are typically phytophilous, whereas larger species are terricoles (Yang and Chen 1997). The vegetation of the temperate meadow steppes is mainly composed of one or two‐year‐old and perennial herbs, with high vegetation cover. Endemic species such as *C. albomarginatus* and *C. aethalinus*, because of their small size, can easily climb onto these herbs (Yang and Chen 1997). *A. fusca fusca* is moderately large, and belongs to the terricoles biotype with a phytophilous tendency, and is closely correlated with dense vegetation cover and is a species of the mountain meadows (Yang and Chen 1997). This study demonstrated that 42% of grasshopper species were distributed in temperate meadow steppe and temperate desert steppe following short‐term grazing exclusion, whereas 19% occurred in mountain meadows. Compared to post‐exclusion studies in the *Leymus chinensis* steppe of Inner Mongolia, grasshopper abundance increased by 24%, with community structure shifts showing a decline in ground‐dwelling species such as *Dasyhipps barbipes* and *Myrmeleotettix palpalis*, but an increase in foliage‐dwelling species like *Chorthippus fallax* and *Omocestus haemorrhoidalis* (Qiu and Li 1997). From the temperate desert steppes to the mountain meadows, vegetation cover presented a “decreasing‐increasing” trend, and the number of locusts and grasshoppers per quadrat showed a “decreasing‐increasing‐decreasing” trend.

In contrast to the results of the same study area 40 years ago, this study showed that some locust and grasshopper species shifted toward higher altitudes, causing changes in the community structure of dominant locusts and grasshoppers at higher altitudes. For example, 40 years ago, *S. scalaris scalaris* was concentrated within the altitude range of 1100–2400 m (Huang and He 1984), whereas *C. italicus* was mainly distributed within the altitude range of 900–2100 m (Chen 1981). *O. caerulescens* mainly existed within the altitude range of 800–1800 m (Chen 1981; Huang and He 1984), whereas *G. sibiricus* was found within the altitude range of 1800–2700 m (Chen 1981; Huang and He 1984). This study found that *S. scalaris scalaris* had shifted to the altitude range of ≥ 1800 m and although *C. italicus* was found at each altitude previously, it is currently concentrated within the altitude range of 1000–2200 m. *O. caerulescens* shifted to altitudes of 1800–2100 m, whereas *G. sibiricus* shifted to altitudes above 1900 m. Moreover, with its place as a high‐altitude dominant species being taken by *S. scalaris scalaris* , *G. sibiricus* became a common species, although further research is needed to clarify and confirm its succession mechanisms and patterns. Interestingly, 40 years ago, *P. microptera microptera*, the indicator species for high altitudes, was concentrated within the altitude range of 2700 m (Chen 1981; Fan 2011). However, in this survey, it appeared only within the altitude range of 1000–1800 m and showed a shift toward lower altitudes. The reason behind this, however, still needs further clarification.

According to this research, temperature plays a crucial role in determining the distribution of dominant locusts and grasshoppers at different altitudes and latitudes, whereas precipitation primarily affects their distribution in different types of grasslands. The contribution degrees of temperature and precipitation exceeded those of other factors, validating H2. Temperature can directly or indirectly affect the growth and development of locusts and grasshoppers as well as their host plants (Jonas and Joern 2007). Locusts and grasshoppers usually inhabit dry and barren habitats with a high surface temperature and low vegetation cover (Sliacka et al. 2013). As expected, the results of the field survey showed that the number of locusts and grasshoppers was closely related to vegetation cover, with 59% of the dominant species distributed in temperate steppes, temperate deserts, and temperate desert grasslands with low vegetation cover and high surface temperature.

### 4.2 Indicator Value (IndVal) Analysis

The results of the Indval analysis were only partially consistent with the field survey results, and H3 was not validated. The theoretical calculation results were consistent with the field survey results in relation to the indicator species in the mountainous meadows and temperate deserts, that is, *S. scalaris scalaris* and *D. tartarus*, respectively. The inconsistent results involved two scenarios. In the first scenario, the Indval analysis failed to identify an indicator species, where the theoretical calculation results revealed no indicator species for the temperate meadow steppes, the temperate steppes, or the temperate steppes. However, in the field survey results, *Omocestus* and *Chorthippus* species served as indicator species for the temperate meadow steppes, whereas *O. decorus decorus* was the indicator species for the temperate steppes, and *C. italicus* and *D. kraussi kraussi* emerged as indicator species for the temperate desert steppes (Chen 2001; Huang and He 1984).

In the second scenario, the IndVal analysis identified indicator species which were not found in the field survey. For example, according to the IndVal analysis, the indicator species for the temperate deserts included *D. kraussi kraussi*, *O. ventralis* , and *C. coelesyriensis*, whereas *O. decorus decorus* was the indicator species for the low altitudes, and *O. d. decorus* and *C. barbarous barbarous* were indicator species for the intermediate altitudes. The indicator species for the intermediate and high latitudes were *S. scalaris scalaris* and *O. miniata* , respectively. However, these results were inconsistent with the field survey results (Arimoro and Keke 2021). Therefore, this study concluded that the IndVal analysis is not entirely suitable for assessing the applicability of locusts and grasshoppers as indicator species for grassland restoration within the short‐term enclosure mode. The reasons are as follows: (1) This method is mainly applied to indicators for the restoration of aquatic ecosystems and sewage. Existing reports on the application of this method to Orthoptera mostly involve the use of Orthoptera as a sensitive indicator for grassland habitat structure under land use change (Zhang, Fan, et al. 2022; Zhang, Wen, et al. 2022; Zhao et al. 2019). However, this indicator is susceptible to land use change (abandonment or intensification) and climate change (Szanyi et al. 2021). (2) This method combines the average abundance and probability of occurrence of a species within a quadrat group as the indicator value of the species, making it necessary to define the quadrat group combination for the data. Because of the increasing number of groups used by species, there will be a decrease in the species indicator values (Dufrêne and Legendre 1997), for which the theoretical calculation results may not be realistic.

## 5 Conclusions

Under short‐term grazing exclusion, grasshopper diversity varied significantly across grassland types. From temperate desert steppe to mountain meadows, as vegetation coverage and altitude increased and latitude decreased, grasshopper abundance similarly displayed a “decrease–increase–decrease” trajectory. Concurrently, the community structure of locusts and grasshoppers shifted from a decrease in terricole species such as *Sphingonotus* to an increase in phytophilous species like *O. haemorrhoidalis* , *P. microptera microptera*, and *O. petraeus*. Mid‐latitude and mid‐altitude regions, where grasshopper diversity is more susceptible to environmental stressors (e.g., climate fluctuations) and anthropogenic activities (e.g., grazing pressure), should be prioritized as critical zones for grassland restoration policies. Notably, over the past 40 years, species *G. sibiricus* , *S. scalaris scalaris* , *O. caerulescens* , and *C. italicus* shifted toward higher altitudes, altering high‐altitude community dynamics. For instance, *S. scalaris scalaris replaced G. sibiricus* as the dominant species at high elevations. These species should be key monitoring targets for future high‐altitude grassland restoration efforts.

The diversity and abundance of locusts and grasshoppers varied across the different grassland types within short‐term enclosure mode. From the temperate desert steppes to the mountain meadows, vegetation cover presented a “decreasing‐increasing” trend, and the number of locusts and grasshoppers per quadrat showed a “decreasing‐increasing‐decreasing” trend. As a result of increased vegetation cover and altitude and decreased latitude, the community structure of locusts and grasshoppers shifted from a decrease in terricoles species such as *Sphingonotus* to an increase in phytophilous species like *O. haemorrhoidalis* , *P. microptera microptera*, and *O. petraeus*. The combined effects of environmental pressures and human disturbances make mid‐latitude and mid‐altitude regions hotspots requiring targeted intervention in grassland restoration strategies. In contrast to the results of the study area 40 years ago, this survey showed that *G. sibiricus* , *S. scalaris scalaris* , *O. caerulescens* , and *C. italicus* shifted toward higher altitudes, causing changes in the community structure of locusts and grasshoppers at higher altitudes; *S. scalaris scalaris* replaced *G. sibiricus* as a high‐altitude dominant species, and *P. microptera microptera* shifted toward lower altitudes.

## Author Contributions

**Chuanen Li:** data curation (lead), formal analysis (lead), investigation (equal), methodology (lead), writing – original draft (lead), writing – review and editing (equal). **Xingmin Song:** investigation (equal). **Mengjia Wang:** investigation (equal). **Roman Jashenko:** supervision (lead). **Jun Lin:** supervision (lead). **Zhujun Cao:** investigation (equal). **Huixia Liu:** investigation (equal). **Rong Ji:** funding acquisition (lead), supervision (equal), validation (lead), writing – review and editing (equal).

## Funding

This project was supported by the Natural Science Foundation of Xinjiang Uygur Autonomous Region (Project No. 2023D01D08); Tianshan Talent Program of Xinjiang Uygur Autonomous Region (Project No. TSYCLJ0016); the National Natural Science Foundation of China (Project No. 32260254); Project number 2022xjkk0600 “Investigation and Assessment of Natural Disasters in the Ili River Basin” is a national science and technology basic work special project under the “Third Xinjiang Comprehensive Scientific Expedition” special plan of the Ministry of Science and Technology, with a support fund of 15 million yuan and an implementation period from 2022 to 2025; the National Key Research and Development Program of China (Project No. 2022YFD1400505).

## Ethics Statement

The authors have nothing to report.

## Consent

All the authors agreed to publish this paper.

## Conflicts of Interest

The authors declare no conflicts of interest.

## Supporting information


**Data S1:** ece372704‐sup‐0001‐DataS1.zip.

## Data Availability

The data that support the findings of this study are available in the Supporting Information of this article.

## Appendix 1

**TABLE A1:** Meteorological stations in the study area.

Station	Latitude	Longitude	Altitude	Name
51,328	44.2	80.42	774	Khorgos
51,329	44.05	80.85	641	Huocheng County
51,431	43.95	81.33	664.3	Yining City
51,434	43.97	81.53	771	Yining County
51,430	43.85	81.15	601	Chabuchar County
51,437	43.15	81.13	1854.6	Zhaosu County
51,438	43.18	81.77	1210.9	Tekes County
51,435	43.47	82.23	775.6	Gongliu County
51,433	43.8	82.57	1106.1	Nilek County
51,436	43.45	83.3	929.1	Xinyuan County

**TABLE A2:** Indicator values and significance of indicator species under different grassland types.

Locusts and grasshoppers species	Montane meadow	Temperate meadow steppe	Warm desert	Warm desert steppe	Warm steppe	*P* value
*Chorthippus albomarginatus*	0	0.069	0	0	0	0.298
*Chorthippus aethalinus*	0	0.023	0	0	0	0.486
*Oedaleus decorus decorus*	0.016	0.141	0.024	0.26	0.259	0.179
*Calliptamus barbarous barbarous*	0.005	0.02	0.082	0.178	0.044	0.238
*Oedipoda miniata*	0	0.024	0.027	0.187	0.092	0.228
*Omocestus haemorrhoidalis*	0.231	0.172	0	0	0.006	0.106
*Dociostaurus kraussi kraussi*	0.004	0.002	0.286	0.029	0	0.02*
*Omocestus ventralis*	0	0.001	0.281	0.028	0	0.011*
*Acrida oxycephala*	0	0	0	0.005	0.091	0.133
*Sphingonotus savignyi*	0	0.015	0	0	0	0.625
*Oedaleus infernalis saussure*	0	0.005	0	0.024	0	0.659
*Oedipoda caerulescens*	0.004	0.049	0.025	0.096	0.011	0.54
*Dericorys tibialis*	0	0	0	0.009	0	0.493
*Dociostaurus tartarus*	0.001	0.007	0.431	0.091	0.065	0.01*
*Sphingonotus coerulipes*	0.001	0.003	0.074	0.059	0.01	0.353
*Sphingonotus salinus*	0	0.01	0	0.009	0	0.999
*Omocestus viridulus*	0	0.115	0	0	0	0.121
*Omocestus petraeus*	0.152	0.014	0	0	0	0.066
*Ramburiella turcomana*	0	0	0.043	0.044	0	0.328
*Pyrgodera armata*	0	0	0.189	0.001	0	0.015*
*Arcyptera fusca fusca*	0.095	0	0	0	0	0.101
*Calliptamus coelesyriensis*	0	0.002	0.337	0.012	0	0.009**
*Gomphocerus sibiricus*	0.086	0.003	0	0	0	0.152
*Pararcyptera microptera microptera*	0.04	0.003	0	0.001	0	0.335
*Notostaurus albicornis*	0	0	0	0.065	0	0.263
*Chrysochraon dispar major*	0	0.01	0	0.003	0	0.905
*Sphingonotus nebulosus*	0	0.001	0	0.068	0	0.226
*Chorthippus biguttulus*	0	0.006	0	0.011	0	0.897
*Calliptamus italicus*	0.021	0.096	0.166	0.184	0.211	0.441
*Egnatius apicalis*	0	0.022	0	0.001	0	0.54
*Conophyma zhaosuensis*	0.031	0.024	0	0	0	0.709
*Stauroderus scalaris scalaris*	0.402	0.097	0	0	0.002	0.008**

* Indicates a significant impact (*p* < 0.05).

** Indicates an extremely significant impact (*p* < 0.01), same below.

**TABLE A3:** Species indicator values and their significance under different altitudes.

Locusts and grasshoppers species	High altitude	Low altitude	Mid altitude	*p*
*Chorthippus albomarginatus*	0.127	0	0.001	0.001**
*Chorthippus aethalinus*	0	0	0.021	0.315
*Oedaleus decorus decorus*	0.005	0.279	0.347	0.009**
*Calliptamus barbarous barbarous*	0	0.389	0.065	0.001**
*Oedipoda miniata*	0	0.489	0.036	0.001**
*Omocestus haemorrhoidalis*	0.500	0	0.062	0.001**
*Dociostaurus kraussi kraussi*	0	0.039	0.081	0.181
*Omocestus ventralis*	0.02	0.002	0.05	0.268
*Acrida oxycephala*	0	0.023	0.001	0.157
*Sphingonotus savignyi*	0	0.025	0	0.103
*Oedaleus infernalis saussure*	0	0.014	0.013	0.762
*Oedipoda caerulescens*	0	0.079	0.119	0.152
*Dericorys tibialis*	0	0	0.007	1
*Dociostaurus tartarus*	0	0.195	0.136	0.019*
*Sphingonotus coerulipes*	0	0.139	0.008	0.002**
*Sphingonotus salinus*	0	0.002	0.024	0.3
*Omocestus viridulus*	0.227	0	0.001	0.001**
*Omocestus petraeus*	0.091	0	0.004	0.008**
*Ramburiella turcomana*	0	0.004	0.029	0.295
*Pyrgodera armata*	0	0.008	0.005	0.897
*Arcyptera fusca fusca*	0.019	0	0.001	0.222
*Calliptamus coelesyriensis*	0	0.011	0.080	0.038*
*Gomphocerus sibiricus*	0.076	0	0.002	0.004**
*Pararcyptera microptera microptera*	0	0	0.041	0.082
*Notostaurus albicornis*	0	0.017	0.015	0.754
*Oedaleus decorus asiaticus*	0	0	0.021	0.308
*Sphingonotus nebulosus*	0	0.058	0.003	0.069
*Chorthippus biguttulus*	0.005	0.004	0.006	0.965
*Calliptamus italicus*	0.035	0.165	0.414	0.001**
*Egnatius apicalis*	0.036	0.001	0	0.061
*Conophyma zhaosuensis*	0.182	0	0	0.001**
*Stauroderus scalaris scalaris*	0.466	0	0.033	0.001**

* Indicates a significant impact (*p* < 0.05).

** Indicates an extremely significant impact (*p* < 0.01), same below.

**TABLE A4:** Indicator values and significance of indicator species under different latitudes.

Locusts and grasshopper species	High latitude	Low latitude	Mid latitude	*p*
*Chorthippus albomarginatus*	0	0.036	0.016	0.238
*Chorthippus aethalinus*	0	0	0.02	0.337
*Oedaleus decorus decorus*	0.244	0.066	0.273	0.117
*Calliptamus barbarous barbarous*	0.203	0.004	0.129	0.02*
*Oedipoda miniata*	0.323	0	0.077	0.001**
*Omocestus haemorrhoidalis*	0.012	0.472	0.03	0.001**
*Dociostaurus kraussi kraussi*	0.091	0	0.056	0.114
*Omocestus ventralis*	0.017	0	0.055	0.213
*Acrida oxycephala*	0.041	0	0	0.025*
*Sphingonotus savignyi*	0	0	0.013	0.608
*Oedaleus infernalis saussure*	0	0	0.04	0.112
*Oedipoda caerulescens*	0.007	0.033	0.168	0.012*
*Dericorys tibialis*	0	0	0.007	1
*Dociostaurus tartarus*	0.122	0	0.2	0.011*
*Sphingonotus coerulipes*	0.192	0	0.003	0.001**
*Sphingonotus salinus*	0.01	0	0.007	0.888
*Omocestus viridulus*	0	0.001	0.087	0.022*
*Omocestus petraeus*	0	0.028	0.021	0.511
*Ramburiella turcomana*	0	0	0.047	0.076
*Pyrgodera armata*	0	0	0.02	0.338
*Arcyptera fusca fusca*	0	0.018	0.001	0.187
*Calliptamus coelesyriensis*	0.002	0	0.101	0.015*
*Gomphocerus sibiricus*	0.046	0	0.002	0.064
*Pararcyptera microptera microptera*	0.001	0.03	0.004	0.212
*Notostaurus albicornis*	0.054	0	0.003	0.036*
*Chrysochraon dispar major*	0	0	0.02	0.369
*Sphingonotus nebulosus*	0.001	0	0.054	0.117
*Chorthippus biguttulus*	0.003	0	0.015	0.489
*Calliptamus italicus*	0.21	0.12	0.239	0.58
*Egnatius apicalis*	0.04	0	0	0.035*
*Conophyma zhaosuensis*	0	0	0.067	0.029*
*Stauroderus scalaris scalaris*	0.048	0.259	0.025	0.001**

* Indicates a significant impact (*p* < 0.05).

** Indicates an extremely significant impact (*p* < 0.01), same below.
